# Normal-Phase HPLC-ELSD to Compare Lipid Profiles of Different Wheat Flours

**DOI:** 10.3390/foods10020428

**Published:** 2021-02-16

**Authors:** Sara Melis, Imogen Foubert, Jan A. Delcour

**Affiliations:** 1 Laboratory of Food Chemistry and Biochemistry (LFCB) and Leuven Food Science and Nutrition Research Centre (LFoRCe), KU Leuven, Kasteelpark Arenberg 20 Box 2486, B-3001 Leuven, Belgium; jan.delcour@kuleuven.be; 2 Research Unit of Food & Lipids and Leuven Food Science and Nutrition Research Centre (LFoRCe), KU Leuven Kulak, Etienne Sabbelaan 53, B-8500 Kortrijk, Belgium; imogen.foubert@kuleuven.be

**Keywords:** high-performance liquid chromatography, evaporative light scattering detection, non-linear response, calibration curve, tripalmitin, trilinolein, near-isogenic wheat lines, fatty acid composition

## Abstract

Normal-phase high-performance liquid chromatography (HPLC) is widely used in combination with evaporative light scattering detection (ELSD) for separating and detecting lipids in various food samples. ELSD responses of different lipids were evaluated to elucidate the possibilities and challenges associated with quantification by means of HPLC-ELSD. Not only the number and type of polar functional groups but also the chain length and degree of unsaturation of (free or esterified) fatty acids (FAs) had a significant effect on ELSD responses. Tripalmitin and trilinolein yielded notably different ELSD responses, even if their constituting free FAs produced identical responses. How FA structure impacts ELSD responses of free FAs is thus not predictive for those of triacylglycerols and presumably other lipids containing esterified FAs. Because ELSD responses of lipids depend on the identity of the (esterified) FA(s) which they contain, fully accurate lipid quantification with HPLC-ELSD is challenging and time-consuming. Nonetheless, HPLC-ELSD is a good and fast technique to semi-quantitatively compare the levels of different lipid classes between samples of comparable FA composition. In this way, lipid profiles of different flours from near-isogenic wheat lines could be compared.

## 1. Introduction

There is no widely accepted definition for the term “lipids” as it needs to cover an extremely broad variety of natural compounds. The following definition put forward by Christie in 1987 is still accepted by the American Oil Chemists’ Society [1,2]: *‘Lipids are fatty acids (FAs) and their derivatives, and substances related biosynthetically or functionally to these compounds’*. In naturally occurring lipids (of plant and animal origin), FAs are mostly esterified to glycerol or other alcohols (such as cholesterol) or linked by amide bonds to long-chain bases (sphingoids or bases thereof) or, exceptionally, to other amines. They may also contain carbohydrates, phosphoric groups and/or organic bases [2,3]. Simple lipids such as the glycerol esters of FAs are composed of only two different types of structural moieties, whereas complex lipids have more than two types of structural moieties. This is, e.g., the case for phospho- and galactolipids [3,4]. Based on the presence of alkyl moieties other than FAs in their structure, lipids are subdivided in classes. Many of these contain lots of lipid species differing in (esterified) FA composition. Trilinolein and tripalmitin, e.g., both belong to the class of the triacylglycerols (TAGs).

Despite the emerging success of mass spectrometry (as an important tool in lipidomics) [5,6,7,8,9,10], traditional chromatographic techniques remain widely used for food lipid analysis [11,12,13,14,15,16,17]. Normal-phase high-performance liquid chromatography (HPLC) coupled to evaporative light scattering detection (ELSD) is particularly useful for separating lipids into individual classes according to the number and type of polar functional groups and has successfully been used to study the lipid composition of samples of various origin [18,19,20,21,22,23,24,25]. In ELSD, the HPLC column effluent is nebulized in a stream of air or nitrogen to form an aerosol. Next, the solvent is evaporated in a heating chamber whereas the non-volatile solute particles pass through a light beam, which is reflected and refracted. The scattered light is collected and transformed into a current which relates to the amount of material in the effluent [2]. Unfortunately, in ELSD there is a sigmoidal instead of a linear response between signal and solute concentrations which finds its origin in concentration-dependent changes in the particle size distribution of the aerosol [26,27]. Furthermore, the response depends on the refractive index and density of the solute and may vary twofold as these properties change [28]. Different lipid classes indeed give very different ELSD responses [21,22,24,29,30,31]. Strangely enough, it is often reported that the chain length and degree of unsaturation of the acyl constituents do not appear to have a significant effect on ELSD response and, thus, that different lipid species of the same lipid class generate identical responses [29,32]. 

Gerits and coworkers [33] developed a single run HPLC-ELSD method for both non-polar and polar lipid classes in wheat flour and dough. They used a monolithic silica column and a quaternary gradient of mobile phases which were based on the method of Graeve and Janssen [31] for separating and quantifying a broad range of lipid classes of marine zooplankton. As lipid classes present in cereals differ from those present in marine zooplankton, the composition of the mobile phases as well as their gradient profile was adapted to allow separation of all wheat lipid classes in a single run [33]. Furthermore, a detector allowing altering the signal gain along the run was used so that wheat lipids could not only be separated but also detected in a single run [34]. With their novel method, Gerits and coworkers studied the changes in lipid distribution during bread dough development [33] and the role of lipids in bread making such as affected by lipase use [34]. The same method has been used successfully to investigate the lipid composition of (un)treated wheat milling fractions [35,36,37,38], wheat starch [39] and gluten [40], wheat dough [41,42], cake batter [43] and wheat, rye, barley and oat dough liquor [44,45]. Moreover, it served as a base for HPLC-ELSD methods for analyzing lipid classes in mammalian (heart, liver and brain), vegetable (soybean and wheat) as well as microbial (yeast and bacteria) lipid samples [46,47]. 

The aim of the present study was to study the potential and challenges associated with quantifying lipids by means of HPLC-ELSD. Hereto, ELSD responses of different lipid classes as well as different lipid species belonging to a same lipid class were evaluated. To illustrate the potential of the technique, the HPLC-ELSD method of Gerits and coworkers [33,34] was used to study the lipid profiles of different wheat flours. Wheat flour lipids, although only present in low levels (i.e., typically 2–3%), have important effects in the manufacture of wheat-based products such as bread [3,48]. To unravel the mechanisms whereby they exert these effects, it is indeed important to have appropriate techniques. The authors here chose to use flours from near-isogenic wheat lines (NILs) that differed only in their *Pina-D1* and/or *Pinb-D1* gene(s) [49,50]. Since these NILs are genetically identical except in their puroindoline (PIN) genes, they are valuable tools for research purposes. The obtained insights are however useful in the broad context of ELSD analysis of lipids.

## 2. Materials and Methods

### 2.1. Materials 

Flour from soft wheat (*Triticum aestivum* L.) cultivar Alpowa, three NILs differing in PIN haplotype derived therefrom (PINA null, PINB G46S and PINB W44R), durum wheat (*T. turgidum* L. ssp. *durum*) cultivar Svevo and one soft NIL derived therefrom (Soft Svevo) were as described in Melis and coworkers [42]. Milling and break flour yields as well as flour moisture, ash, protein, damaged starch, PIN, free lipid and bound lipid levels are provided in the same publication [42]. Alpowa wheat has a soft endosperm texture due to the presence of both PIN genes in their wild-type sequences (*Pina-D1a/Pinb-D1a*). The NILs derived from Alpowa have hard endosperm texture due to a mutation in one of their PIN genes [50]. The Svevo durum wheat has a very hard endosperm texture due to the absence of the *Pina-D1* and *Pinb-D1* genes. Soft Svevo was developed through homoeologous transfer of both PIN genes in their wild-type sequences [49].

Lipid standards, i.e., free FAs (FFAs) [dodecanoic (lauric, C12:0), hexadecanoic (palmitic, C16:0), octadecanoic (stearic, C18:0), 9(Z)-octadecenoic (oleic, C18:1), 9(Z),12(Z)-octadecadienoic (linoleic, C18:2), 9(Z),12(Z),15(Z)-octadecatrienoic (linolenic, C18:3) and 4(Z),7(Z),10(Z),13(Z),16(Z),19(Z)-docosahexaenoic (cervonic, C22:6) acids], monoacylglycerols (MAGs; monoolein), diacylglycerols (DAGs; 1,3-dilinolein) and TAGs (tripalmitin and trilinolein) were from Larodan (Solna, Sweden) and had a purity of >99%. Phospholipid and galactolipid standards used for peak identification were as described in Gerits and coworkers [33]. All lipid standards were stored at −80 °C.

All chemicals, solvents and reagents were from Acros Organics (Geel, Belgium; acetic acid, chloroform, hexane and methanol), Chem-Lab (Zedelgem, Belgium; butan-1-ol), Honeywell Riedel-de Haën (Seelze, Germany; propan-2-ol), Merck KGaA (Darmstadt, Germany; acetone, ethyl acetate and isooctane) and Sigma-Aldrich (Bornem, Belgium; acid washed sand, sodium chloride, sulfuric acid, triethylamine and toluene) unless specified otherwise. Solvents for lipid extraction and analysis were HPLC-grade.

### 2.2. ELSD Responses of Different Lipids

FFA, MAG, DAG and TAG lipid standards were dissolved in chloroform at about 5 mg/mL and three times 20 µL thereof was transferred into amber-colored tarred vials. Chloroform was evaporated under a stream of nitrogen and the exact lipid mass (about 100 µg) in each vial was weighed with a Mettler Toledo (Zaventem, Belgium) MT5 Analytical Microbalance. Next, isooctane was added to each vial so that the lipid concentration was exactly 100 µg/mL and samples were vortexed. Lipid standards were analyzed with HPLC-ELSD as described in Gerits and coworkers [33,34]. Briefly, a modular HPLC system (Shimadzu, Kyoto, Japan) with a controller (SCL-10Avp), pump (LC-20AD), autoinjector (SIL-10ADvp) and column oven (CTO-10APvp) set at 40 °C was coupled to an Alltech Model 3300 ELSD (Büchi, Hendrik-Ido-Ambacht, The Netherlands). Detector drift tube temperature was 40 °C, gas flow 1.5 L/min and the impactor mode on. Separation of lipid classes was accomplished with a polar monolithic Chromolith Performance-Si column (100 mm × 4.6 mm inner diameter) and a quaternary gradient of pure isooctane, acetone:ethyl acetate (2:1, *v/v*) containing 70.0 mmol/L acetic acid and propan-2-ol:water (85:15, *v/v*) containing 7.5 mmol/L of both acetic acid and triethylamine. After separation, five min washing with propan-2-ol followed by five min washing with isooctane removed polar impurities and water from the column, prevented pressure fluctuations and re-equilibrated the column for further analyses. The optimized composition and gradient of the mobile phases and alteration of the detector signal gain along the run allowed separation and detection of all wheat flour lipids in a single run [33,34,40]. The total analysis run time was 35 min, with the quaternary gradient, flow rates and detector gains provided in detail in Melis and coworkers [40]. Injection volumes ranged between 0.1 and 25.0 µL. Data was acquired with Shimadzu LCSolution (version 1.23 SP1, Shimadzu, Kyoto, Japan).

### 2.3. Lipid Extraction

Flour non-starch lipids were extracted in triplicate essentially as in Melis and coworkers [40], with the exception that 1.0 g sample (14.0% moisture base) was blended with 28 g acid washed sand prior to extraction. Free and bound non-starch lipids were sequentially extracted with hexane and water-saturated butan-1-ol (WSB), respectively. Total non-starch lipids were directly extracted with WSB. Crude bound and total lipid extracts were purified according to Bligh and Dyer [51] to remove nonlipid material as in Melis and coworkers [40].

### 2.4. Fatty Acid Composition

The FA composition of total non-starch lipids extracted from wheat flour was determined by methylation as in Ryckebosch and coworkers [52]. Briefly, 5.0 mg extracted lipid was dissolved in 1.0 mL toluene. Next, 2.0 mL methanol containing 1.0% sulfuric acid was added and the mixture was kept overnight at 50 °C in a stoppered tube. After addition of 5.0 mL 5.0% sodium chloride, the FA methyl esters (FAMEs) were extracted with 3.0 mL hexane (Sigma–Aldrich, Bornem, Belgium). Following appropriate dilutions, the obtained FAMEs were separated by gas chromatography with cold on-column injection (1.0 μL) and detected with flame ionization detection (Trace GC Ultra, Thermo Scientific, Interscience, Louvain-la-Neuve, Belgium) essentially as in Gheysen and coworkers [53]. An EC Wax column (length 30 m, internal diameter 0.32 mm, film thickness 0.25 μm; Grace, Lokeren, Belgium) was used with the following temperature-time profile: 70–180 °C (10 °C/min), 180–235 °C (4 °C/min), 235 °C (4 min 45 s). 

Standards (Nu-check, Elysian, MN, USA) containing 35 different FAMEs were analyzed for provisional peak identification. Peak areas were quantified with Chromcard for Windows software (Interscience). Internal standard (lauric acid, C12:0) was added to the lipid extract before methylation for quantification of FAMEs. For each lipid extract, a sample lacking internal standard was also analyzed to determine the portion of endogenous lauric acid. FA levels were calculated from the detected levels of FAMEs with conversion factors based on the difference in molecular weight between them.

### 2.5. Lipid Composition

To free and bound non-starch lipids extracted from 1.0 g wheat flour (14.0% moisture base, Section 2.3), 1.0 mg cholesterol (Larodan, Solna, Sweden) was added as internal standard. Cholesterol is a naturally occurring lipid of animal origin and absent in wheat. While wheat flour may contain minor levels of plant sterols such as campesterol [54], their signal did not interfere with that of cholesterol [33]. Lipids were then dissolved in 1.0 mL isooctane and analyzed with HPLC-ELSD as described in Section 2.2. Injection volumes were 0.5 µL for free and 4.0 µL for bound lipids. Lipid levels are presented as the areas under the curve relative to the area under the curve of the internal standard.

### 2.6. Statistical Analyses

Linear, power and polynomial trend lines were fitted to the obtained data and the corresponding equations and R-squared values were obtained with Excel 2016 (Microsoft, Redmond, WA, USA). For several variables, it was verified whether mean values differed significantly using one-way ANOVA with JMP Pro 14 (SAS Institute, Cary, NC, USA). When variables were significantly different (*p* < 0.05), means were further compared using a post-hoc Tukey-Kramer test with a significance level (α) of 0.05.

## 3. Results

### 3.1. ELSD Responses of Different Simple Lipid Classes

**Figure 1:**
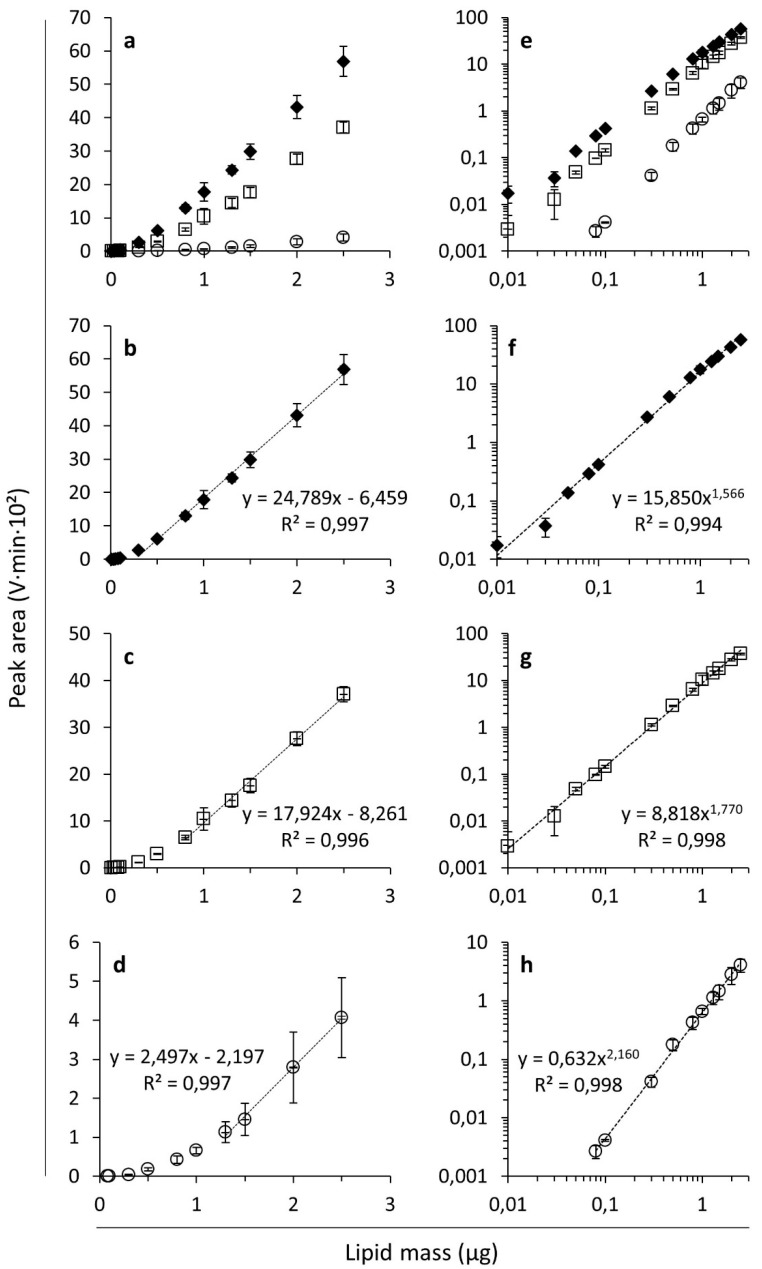
Evaporative light scattering detection responses expressed as peak areas (V·min·10^2^) as a function of the analyzed lipid mass (µg) for free fatty acids (FFAs ◆; linoleic acid), monoacylglycerols (MAGs ○; monoolein) and diacylglycerols (DAGs □; 1,3-dilinolein) on linear (**a**–**d**) and logarithmic (**e**–**h**) scales. Average values with corresponding standard deviations of triplicate measurements are shown. Linear trend lines were fitted to the data in the range of linear response for FFAs (**b**), DAGs (**c**) and MAGs (**d**). Power trend lines were fitted to the data for FFAs (**f**), DAGs (**g**) and MAGs (**h**). The graphs show the corresponding trend line equations and R-squared values.

Figure 1a,e display the ELSD peak areas as a function of mass for FFAs, MAGs and DAGs on a linear and a logarithmic scale, respectively. In line with earlier findings [24,29,31], the ELSD response (i.e., peak area/lipid mass) varied with the lipid class. In the present case, as also observed by Jones and coworkers [22] and Donot and coworkers [21], the ELSD responses decreased in the order FFAs, DAGs and MAGs and thus were not related to lipid molecular weight.

As reported earlier [26,27], the lipid response curves were sigmoidal when plotted on linear axes (Figure 1a) making it possible to define a range over which the investigated lipids exert a linear response. This range depended on the lipid class under consideration. For example, the response of FFAs was successfully fitted to a linear function in the range of 0.3–2.5 µg FFAs (Figure 1b), DAGs in the range of 0.8–2.5 µg (Figure 1c), and MAGs only in a range of 1.3–2.5 µm (Figure 1d). Indeed, the concentration range of linear response is related to the intensity of the ELSD response of the lipids investigated. For example, MAGs have a low ELSD response and therefore, the range of linear behavior is observed at higher lipid masses. Linear fits have been applied before for modeling ELSD responses of lipids within a particular lipid mass range [21,32] and responses at lower lipid levels have been reported not to be linear [22,29]. When plotted on a logarithmic scale, the responses are fairly linear with some flattening at high lipid masses (Figure 1e). It follows that the non-linear response characteristic of ELSD is well fitted by power trend lines (Figure 1f–h), which has been described before for various lipid classes [24,27,55]. Not only linear and power trend lines, but also polynomial curves have been applied to model the dose-ELSD response relationship of lipids [22,47] (see also Section 3.2). The response has also been described as exponential in certain lipid mass ranges [28], but this was not satisfying in our case.

### 3.2. ELSD Responses of Different Simple Lipid Species Belonging to the Same Lipid Class

**Figure 2:**
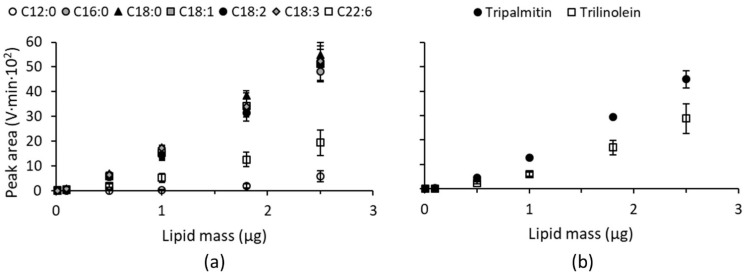
Evaporative light scattering detection responses expressed as peak areas (V·min·10²) as a function of the analyzed lipid mass (µg) for different (**a**) free fatty acids and (**b**) triacylglycerols. Average values with corresponding standard deviations of triplicate measurements are shown. C12:0, lauric acid; C16:0, palmitic acid; C18:0, stearic acid; C18:1, oleic acid; C18:2, linoleic acid; C18:3, linolenic acid; C22:6, cervonic acid.

Figure 2 shows the dose-ELSD response relationships of a number of different FFAs and TAGs varying in FA composition. Responses of palmitic (C16:0), stearic (C18:0), oleic (C18:1), linoleic (C18:2) and linolenic (C18:3) acids were comparable and markedly higher than those of lauric (C12:0) and cervonic (C22:6) acids (Figure 2a). Of the latter two, the response of lauric acid was the lowest. Christie [29] reported some loss of FFAs at higher temperatures due to evaporation in the detector. However, it is very unlikely that this occurred under the present experimental conditions since the temperature in the detector drift tube was only 40 °C whereas the boiling points of the investigated FFAs exceeded 300 °C (Table 1). Therefore, the low detector responses of lauric and cervonic acids cannot be explained by evaporation of these FFAs in the detector.

Regarding TAGs varying in FA composition, the response of tripalmitin was notably higher than that of trilinolein (Figure 2b). This is in contrast with findings of Kobayashi and coworkers [32], who found the responses of these TAGs to be very similar. The opposing results are most likely caused by differences in the applied chromatographic methods as, in general, the mobile phases and oven temperature affect ELSD response [21]. Kobayashi and coworkers [32] also demonstrated that TAGs varying in FA composition do not necessarily have the same detector response as the ELSD responses of trimargarin and tristearin were markedly lower than those of tripentadecanoin, trilinolein and trilinolenin [32].

Since ELSD responses depend on the refractive index and density of the solute [28], we investigated whether these physical properties can explain the differences in response observed for the investigated FFAs and TAGs (Figure 2). The data in Table 1 reveal that the refractive indices of FFAs and TAGs relate quite well to the number of double bonds per FA they contain. Linear regression of refractive index as a function of the number of (up to three) double bonds per FA results in the following equation: y = 0.019x + 1.434 (*R²* = 0.753). From this relation, the refractive indices of cervonic acid and trilinolein, which were not found in available literature sources, are predicted to be 1.548 and 1.472 respectively. The predicted refractive index of cervonic acid is notably higher than values reported for the other investigated FFAs and TAGs (Table 1) and might therefore explain the deviating response of this FFA. It should be noted, however, that the linear relationship applied to predict the refractive index of cervonic acid may not be valid for lipids containing more than three double bonds per FA. The predicted refractive index of trilinolein (1.472) is comparable to the refractive index of lauric acid (1.4183, Table 1) and in the range of refractive indices reported for the FFAs and TAGs with an identical response (Table 1). It can therefore not explain their lower ELSD response. Also for density, a linear relationship with the number of double bonds per FA (y = 0.023x + 0.862; *R²* = 0.570) is obtained when the value of stearic acid is omitted. Based on this linear relationship, the density of cervonic acid is predicted to be 1.000. This is markedly higher than densities of the other investigated FFAs and TAGs (Table 1). But also in this case there may not be a linear relationship for cervonic acid. For lauric acid and trilinolein, the reported densities lie within the range reported for the other investigated FFAs and TAGs (Table 1). Therefore, we believe that for the lipids investigated here differences in ELSD response do not result from differences in refractive index and/or density. ELSD operates essentially in three steps. First the HPLC effluent is nebulized, the mobile phase is then evaporated and finally the residual non-volatile analyte particles are detected by measuring the scattered light. Not the number of analyte particles but the diameter thereof causes the ELSD response to depend on analyte concentration. Moreover, the size, shape and surface properties of the analyte particles determine the interaction between them and the light [56]. It is reasonable to assume that these properties are affected by the structure of the investigated lipids and that not only the number and type of polar functional groups but also the chain length and degree of unsaturation of their FAs has an impact. It is, e.g., well-known that the structure or molecular shape of lipids determines the type of monolayer or liquid-crystal mesophase is which they organize themselves [57,58]. Therefore, the observed differences in ELSD response presumably originated from differences in the size, shape and/or surface properties of the analyte particles formed by lipids of varying structures. 

The dose-response relationships of Figure 2 reveal both non-linear and linear behavior. For lipid masses ranging between 0.5 and 2.5 µg, ELSD responses of the investigated FFAs and TAGs are fairly linear, with the poorest fits for lauric acid and trilinolein (Table 2). These lipids probably exhibit a linear behavior in a higher lipid mass range as they have a relatively low ELSD response and the range of linear response seems to be related to the intensity of the response for simple lipids (Section 3.1). In contrast to what is the case for the dose-response relationships in Figure 1, the responses of the investigated FFAs and TAGs varying in FA composition are fitted only with limited success by power trend lines (Table 2). In this case, higher R-squared values are obtained with a second order polynomial trend line (Table 2). This apparent contradiction is because less and different points were included to construct the dose-response relationships in Figure 2 than for those in Figure 1, which can affect the suitability of a model. Evidently, one needs to consider how many and which points to include in a calibration curve and which model (linear, power, polynomial, exponential) to apply to obtain the best fit. Ideally, a calibration curve should cover a mass range as wide as possible and have sufficient points in between at regular intervals. The most appropriate model for fitting the calibration curve can be selected based on the coefficient of determination or R-squared value. The closer the R-squared value is to one, the better the ELSD response is predicted by the model. Nonetheless, a linear fit can be preferred even if it does not have the highest R-squared value because of its ease of use. It can be particularly useful for routine works or screening purposes where relative comparison between samples is more important than exact absolute quantification.

Finally, Table 2 lists the retention times of all investigated FFAs as well as those of TAGs varying in FA composition. Retention times were comparable for all investigated FFAs, regardless of chain length or degree of unsaturation, whereas TAGs varying in FA composition had slightly but still significantly different retention times (Table 2).

### 3.3. Fatty Acid Composition of Flour from Near-Isogenic Wheat Lines

The predominant FAs present in non-starch lipids of the investigated flours were linoleic (60.9–65.3%), palmitic (19.5–20.4%) and oleic (10.7–14.1%) acids (Table 3). This is well in line with values reported in literature (50–65%, 19–26% and 10–21%, respectively) [3]. Furthermore, minor levels of α-linolenic, stearic and eicosenoic acid were detected. When present in their unesterified form, all these FAs produce comparable ELSD responses (Figure 2a). FFAs released from non-starch wheat flour lipids can thus be quantified by means of a calibration curve prepared from any FFA selected from the group of palmitic, stearic, oleic, linoleic and linolenic acids. This can be particularly useful when monitoring the release of FFAs over time during wheat flour storage (endogenous lipase action) or when treating wheat flour with exogenously added lipases. For other non-starch wheat flour lipids containing esterified FAs, such as TAGs, quantification by means of HPLC-ELSD may result in inaccurate results. Indeed, if the FA composition of the lipid class to be quantified differs from that of the lipid used to prepare a calibration curve, results will be imprecise. An accurate calibration curve can only be prepared if a lipid class is purified from the sample to be investigated so that identical FA composition is ensured.

The FA composition of flours from different wheat cultivars (Alpowa vs. Svevo) differed significantly but was similar for flours from NILs of a same cultivar (Table 3). Flours derived from Alpowa had significantly higher levels of linoleic acid and, concomitantly, significantly lower levels of oleic, α-linolenic and stearic acids than those derived from Svevo. Since all wheats were produced under the same conditions [42], results demonstrate that FA composition of wheat flour is cultivar-dependent. For the applied NILs, differences in their *Pina-D1* and/or *Pinb-D1* gene(s) had no impact on the FA composition of the obtained flours.

### 3.4. Lipid Composition of Flour from Near-Isogenic Wheat Lines

**Figure 3:**
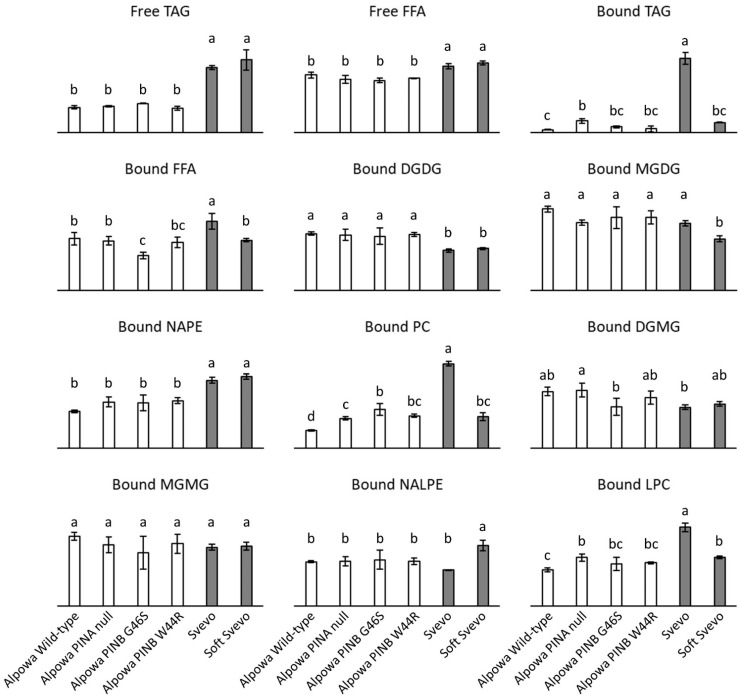
Lipid composition of flours from near-isogenic wheat lines. Lipid levels are presented as the area under the curve relative to the area under the curve of the internal standard. Free and bound non-starch lipids were sequentially extracted with hexane and water-saturated butan-1-ol, respectively. Averages with corresponding standard deviations of triplicate measurements are shown. For each lipid class, bars with differing letters are significantly different from each other (*p* < 0.05). TAG, triacylglycerols; FFA, free fatty acids; DGDG, digalactosyldiacylglycerols; MGDG, monogalactosyldiacylglycerols; NAPE, N-acyl phosphatidylethanolamines; PC, phosphatidylcholines; DGMG, digalactosylmonoacylglycerols; MGMG, monogalactosylmonoacylglycerols; NALPE, N-acyl lysophosphatidylethanolamines; LPC, lysophosphatidylcholines.

The composition of the non-starch lipids extracted from the investigated flours is shown in Figure 3. Lipid class levels were expressed as the areas under the curve relative to that of the internal standard. Since each lipid class produces a different ELSD response (Section 3.1), levels of different lipid classes could not be compared and it was therefore deemed of little use to show y-axes with numerical values in the graphs of Figure 3. Nonetheless, even without converting ELSD responses in lipid class levels (expressed in, e.g., %, = absolute quantification), it is clear that flours from different wheat cultivars (Alpowa vs. Svevo) have significantly different lipid profiles. Also for flours from NILs of the same cultivar, some significant differences are observed. This was most outspoken so for Svevo vs. Soft Svevo. This relative comparison per lipid class among different samples is possible because the investigated wheat flours do not differ (much) in their FA composition (Section 3.3).

In the free lipid fractions, i.e., the lipids extracted with hexane, only TAGs and FFAs were detected (Figure 3). Flour from Svevo and Soft Svevo had significantly more of these free lipids than flour from Alpowa and its NILs. Free TAGs and FFAs in these samples were quantified by using the linear trend line of trilinolein and linoleic acid, respectively (Table 2). When quantifying TAGs, this may have resulted in (slightly) inaccurate results since TAGs differing in their FA composition may produce different ELSD responses. Nonetheless, since linoleic acid is the most abundant FA present in wheat flour (Section 3.3), using the trendline of trilinolein seemed most appropriate. Quantification of FFAs is considered to have been accurate since all FFAs released from non-starch wheat flour lipids produced comparable ELSD responses (Section 3.3). In flour from Alpowa and its NILs, 44–51% of the free lipids were TAGs and 14–17% were FFAs. This is in line with literature stating that wheat flour free lipids are approximately 75% nonpolar and 25% polar lipids [3]. The free polar lipids in flour from Alpowa and its NILs were probably not detected due to their signals being below the detection limit. The free lipids of flour from Svevo and Soft Svevo were for the major part nonpolar with about 70% of them being TAGs and 30% FFAs. Overall, the present findings are much in line with those of Gerits and coworkers [33] who by using a similar approach found that free lipids of flour from Claire had about 42% TAGs and 30% FFAs.

The most predominant wheat flour non-starch lipids reported in literature are TAGs (21–47%), digalactosyldiacylglycerols (DGDGs) (13–17%), monogalactosyldiacylglycerols (5–6%), N-acylphosphatidylethanolamines (NAPEs) (4–5%), and phosphatidylcholines (PCs) (4–6%) [3]. All such lipids were detected in the bound lipid fractions, i.e., the lipids extracted with WSB, of the investigated flours (Figure 3). Furthermore, some minor lipids were detected in the bound lipid fractions. They were the corresponding lysolipids of the most predominant flour lipids as well as FFAs. Bound flour lipids consist almost exclusively of polar galacto- and phospholipids [3]. Quantification of bound TAGs and FFAs in the samples [by using the linear trend line of trilinolein and linoleic acid, respectively (Table 2)] revealed that only 1–3% of the bound lipids were TAGs and that 4–8% were FFAs. This is consistent with TAG and FFA levels found in bound lipids of flour from Claire (about 2% and 3%, respectively) [33]. The majority of the bound lipids in the investigated flour samples were thus indeed polar galacto- and phospholipids. Levels of the major galactolipid DGDG were significantly higher in flours from Alpowa and its NILs than in flours from Svevo and Soft Svevo, which was the other way around for levels of the major phospholipid NAPE (Figure 3). Such findings are relevant when investigating the role of flour lipids in wheat based products since such lipids (especially galactolipids) have beneficial effects on bread quality [3]. For the other most dominant flour non-starch lipids (monogalactosyldiacylglycerols and PCs), differences were not only observed when comparing different cultivars (Alpowa vs. Svevo) but also when comparing NILs of a same cultivar (most obvious for Svevo vs. Soft Svevo). This was likely not directly due to differences in their *Pina-D1* and/or *Pinb-D1* gene(s) but to differences in milling behavior of the wheats and/or endogenous lipase activity. PINs are the major determinants of grain hardness [61]. Although endosperm hardness only has a slight influence on the polar lipid levels present in flour [62], it determines the wheat milling and therefore flour characteristics [63,64]. Remarkably, the levels of PCs in the different flour samples followed the same trend as those of bound TAGs (Figure 3). Flour from Svevo contained most, flour from Alpowa least and the other flours had intermediate levels of these lipids. They presumably originate from oil bodies or spherosomes, spherical structures with a core of TAGs surrounded by a phospholipid monolayer. Such oil bodies are abundantly present in the germ and to a lesser extent in the aleurone layer of wheat [3,65,66,67]. The present results pointed out that these kernel tissues were most abundantly present in flour from Svevo and least in flour from Alpowa, and that bound TAGs and PCs from oil bodies may be good markers for flour contamination by such tissues. Lysophosphatidylcholines (LPCs) were also detected in variable levels in the bound lipids extracted from the investigated flours (Figure 3). LPCs constitute about 85% of the starch lipid fraction of wheat flour [3,68,69]. Starch lipids occur inside starch granules, complexed with amylose, and are therefore not extractable at room temperature [3,69,70]. We argue that when starch granules are damaged in the process of wheat milling, the LPCs present in damaged starch may be (partly) extractable. Hence, LPCs were detected in the bound lipid fractions extracted from all investigated wheat flours and most abundantly in flour from Svevo, a durum wheat with very hard texture and thus high degree of starch damage [63,64].

## 4. Discussion

Investigating the impact of FA composition on ELSD response taught that, for both FFAs and TAGs, variations in (esterified) FA composition result in different ELSD responses (Figure 2). This is not necessarily true in all cases as palmitic, stearic, oleic, linoleic and linolenic acids yield comparable responses. Thus, small variations in chain length and/or saturation ranging from C16:0 until C18:3 do not result in significantly different ELSD responses. However, tripalmitin and trilinolein yielded notably different ELSD responses, even if their corresponding FFAs did not. The way FA structure does or does not impact the ELSD response of FFAs is thus not predictive for the ELSD response of TAGs. It is reasonable to assume that this is not only the case for TAGs but also for other lipids containing esterified FAs such as DGDGs and NAPEs. Although that chain length and degree of unsaturation of acyl constituents do not appear to have a significant effect on ELSD response [29,32], our findings are in agreement with those of other authors who also noticed different ELSD responses for different TAG molecular species [32,71]. Lin [72] reported that ELSD responses of different acylglycerol species can vary by up to 30% while responses of different lipid classes can vary by up to three times. The present results point to even higher differences, with the ELSD response of tripalmitin being more than 50% higher than that of trilinolein and responses of the investigated FFAs containing sixteen or eighteen carbon atoms being about nine times higher than that of lauric acid (Figure 2). Among different lipid classes, ELSD responses of DAGs (1,3-dilinolein)) and FFAs (linoleic acid) were respectively nine and fourteen times higher than that of MAGs (monoolein) (Figure 1).

As ELSD responses depend on the individual FFAs and the FA composition of TAGs and probably other lipids containing esterified FAs, lipid quantification with HPLC-ELSD is challenging. Indeed, lipid classes typically present in food samples contain numerous different species, the (esterified) FA composition of which depends on the origin. González-Thuillier and coworkers [7] identified 72 lipid molecular species in wheat milling and pearling fractions with electrospray ionization tandem triple-quadrupole mass spectrometry. Of these, 12 were FFAs, 11 were DAGs, 10 were TAGs, 9 were DGDGs and 8 were PCs. In buttermilk, over 30 molecular species of PCs, phosphatidylethanolamines, phosphatidylserines and phosphatidylinositols were identified using liquid chromatography/quadruple-time-of-flight mass spectrometry, with PC (16:0/18:1) being the most abundant species [6]. In banana, up to 143 lipid molecular species were detected with liquid chromatography/electrospray ionization tandem triple-quadrupole mass spectrometry. These included 34 TAGs, 24 PCs, 18 DAGs, 16 phosphatidylethanolamines and 15 LPCs [9]. Therefore, it is not possible to use synthesized lipids or lipids purified from a random source as a standard to prepare a calibration curve which represents the response of a lipid class irrespective of the FA composition. Indeed, this could lead to significant overestimations or underestimations since ELSD responses of different molecular species of a same lipid class have been reported to vary with up to 30% [72]. The present results indicated even greater differences (Figure 2). To quantify various lipid classes such as FFAs and TAGs in a sample, purification of these lipid classes from the sample to be investigated such as done by Schaffarczyk and coworkers [47] for, inter alia, DGDGs from wheat flour is necessary to ensure identical FA composition and thus correct calibration curves for each lipid class. Briefly, reference compounds for identification and quantification of lipid classes by HPLC-ELSD first have to be extracted from the sample to be investigated. Next, the obtained crude lipid extract needs to be (pre)fractionated and further separated by consecutive preparative solid-phase extractions and column chromatography separations to obtain pure reference compounds (one for each lipid class of interest). Accurate calibration curves can then be obtained by preparing solutions with different known concentrations of each reference compound and injecting a fixed volume of each solution [47] or by injecting different volumes of a solution containing a known concentration of these reference compounds [33]. Obviously, this is labor intensive as a high degree of purity is required and purified lipids from one sample cannot be used to quantify lipids in another sample with a different FA composition.

Only around 20 FAs occur widely in nature, of which palmitic, oleic and linoleic acids make up about 80% of commodity oils and fats [57]. Also wheat lipids are almost exclusively composed of FAs with sixteen or eighteen carbon atoms and no more than three double bonds (Table 3) [4,73]. Therefore, FFAs in samples where wheat (flour) is the only lipid source can be quantified with HPLC-ELSD based on a calibration curve obtained with any FFA standard selected from the group of palmitic, stearic, oleic, linoleic and linolenic acids. This was demonstrated for FFAs present in flours from different NILs (Section 3.4). In the investigated flours, 8–20% of the total non-starch lipids were FFAs and 20–40% were TAGs. It should be noted that quantification of TAGs may be (slightly) inaccurate since trilinolein was used for calibration as it may have a different ELSD response than the TAGs present in wheat flour. Although previous studies reported much lower proportions of FFAs in wheat flours [4,65], the present results are in line with more recent electrospray ionization tandem triple-quadrupole mass spectrometry analytical data of wheat milling fractions. González-Thuillier and coworkers [7] found that the lipids in different milling fractions of Hereward wheat contained 19–24% FFAs and 10–36% TAGs. Min and coworkers [74] reported that lipids of flours from six wheat lines grown at three levels of nitrogen supply contained on average 31% FFAs and 23% TAGs. For samples with a more different FA composition like coconut and palm kernel oil, being good sources of lauric acid, or fish oil, containing considerable levels of (esterified) cervonic acid [57], quantification of FFAs is not possible with just any synthetic or purified lipid standard. In those cases, lipids have to be extracted and FFAs have to be further purified (e.g., by solid-phase extraction) after which they can be quantified by gas chromatography and flame ionization detection [75] and/or used as a standard to prepare a calibration curve for HPLC-ELSD quantification [47]. Alternatively, mass spectrometry detection, whether or not preceded by normal- or reversed-phase HPLC, can be used to quantify FFAs [76,77,78].

When absolute quantification is not required and the goal is to compare lipid class levels between different samples with a comparable FA composition, HPLC-ELSD is a good and fast technique to perform semi-quantitative analyses of lipid samples. It was here used to compare the lipid profiles of flours from different NILs, which was discussed in depth in Section 3.4. The technique can also be used to evaluate the impact of lipases on the lipid population of a food sample [34,40,41,42]. Such lipases can be endogenously present and/or exogenously added. In that way, semi-quantitative HPLC-ELSD analysis can yield valuable information about the impact of wheat flour aging or added lipases in wheat flour dough.

## 5. Conclusions

ELSD responses not only vary among different lipid classes but also among different molecular species of a same lipid class. As the response depends on the FA composition, lipid quantification with HPLC-ELSD is challenging. Synthesized lipids or lipids purified from a random source cannot be used as standards for constructing calibration curves. Accurate quantification can only be accomplished provided that the FA composition of the lipid used for calibration is identical to that of the lipid to be quantified. To ensure identical FA composition, lipid classes such as FFAs, TAGs, DGDGs or NAPEs need to be purified from the sample to be investigated. Palmitic, stearic, oleic, linoleic and linolenic FFAs generate similar ELSD responses. Therefore, FFAs in samples such as wheat flour containing mainly only such FAs can be quantified correctly with HPLC-ELSD by using a calibration curve prepared from one of these FFAs. ELSD of lipids produces a non-linear response to mass. When preparing calibration curves, it is necessary to evaluate on a case-by-case base which model is best applied to reach the stated goal. When absolute quantification is not required and the aim is to compare lipid class levels between different samples of comparable FA composition, normal-phase HPLC-ELSD is a good and fast technique to perform semi-quantitative lipid analyses of various food samples such as wheat flour.

## Figures and Tables

**Table 1:** Molecular weights and physical properties of free fatty acids (FFAs) and triacylglycerols (TAGs) retrieved from the CRC Handbook of Chemistry and Physics [59] and *ChemSpider* [60]. *ChemSpider* is an online structure database providing search access to hundreds of data sources. The original sources of data collection are indicated with a superscript letter.

Lipid		Molecular Weight (g/mol)	Boiling Point * (°C)	Refractive Index	Density (g/cm³)
FFAs	Lauric acid	200.32	319 ^e^; 331 ^a^	1.4183 °	0.868 °; 0.883 ^a,e^
	Palmitic acid	256.42	351 °; 373 ^c^; 391 ^a^	1.4273 ^a^ 1.4335 °	0.853 °^,a^
	Stearic acid	284.48	371 °; 413 ^a^	1.4299 °	0.941 °^,a^
	Oleic acid	282.46	360 °; 390 ^a^; 468–469 ^b^	1.4582 °^,a^	0.887 ^a^; 0.894 °
	Linoleic acid	280.45	408 ^a^	1.4699 °^,a^	0.902 °^,a,e^
	Linolenic acid	278.43	/	1.4800 °	0.916 °^,d^
	Behenic acid	340.58	440 ^a^	1.4270 °	0.822 °
	Erucic acid	338.57	457 ^a^	1.4758 °	0.860 °
	Cervonic acid	328.49	/	/	/
TAGs	Tripalmitin	807.32	624 °	1.4381 °	0.875 °
	Tristearin	891.48	/	1.4395 °	0.856 °
	Triolein	885.43	409–416 ^c^	1.4676 °	0.915 °
	Trilinolein	879.38	/	/	0.925 ^d^

* at 101,325 Pa (≈atmospheric pressure); /, property was not found; ° Haynes and coworkers [59]; ^a^ Alfa Aesar (https://www.alfa.com (accessed on 7 January 2021)); ^b^ Food and Agriculture Organization of the United Nations (http://www.fao.org/food/food-safety-quality/scientific-advice/jecfa/jecfa-flav (accessed on 7 January 2021)); ^c^ LabNetwork (https://www.labnetwork.com (accessed on 7 January 2021)); ^d^ Sigma-Aldrich (https://www.sigmaaldrich.com (accessed on 7 January 2021)); ^e^ SynQuest (http://synquestlabs.com (accessed on 7 January 2021)).

**Table 2:** Equations and R-squared values of linear, power and polynomial trend lines fitted to the evaporative light scattering detection response as a function of the analyzed lipid mass for different free fatty acids (FFAs) and triacylglycerols (TAGs) (Figure 2). Also listed are the retention times of the FFAs and TAGs. Column values with differing letters are significantly different from each other (*p* < 0.05).

Lipid		Linear Trend Line ^a^		Power Trend Line		Polynomial Trend Line		Retention Time ^b^ (min)
		Equation	*R²*	Equation	*R²*	Equation	*R²*	
FFAs	Lauric acid	y = 2.787x – 2.020	0.888	y = 0.585x^0.855^	0.647	y = 1.419x² - 1.386x + 0.182	0.992	9.38 ± 0.02 ^A^
	Palmitic acid	y = 21.517x – 5.520	0.999	y = 14.342x^1.185^	0.983	y = 2.215x² + 14.363x − 0.894	0.998	9.40 ± 0.05 ^A^
	Stearic acid	y = 24.384x – 5.961	0.999	y = 16.889x^1.150^	0.986	y = 2.447x² + 16.513x − 0.913	0.998	9.39 ± 0.02 ^A^
	Oleic acid	y = 22.912x – 6.591	0.998	y = 14.974x^1.177^	0.987	y = 3.126x² + 13.148x − 0.679	0.999	9.39 ± 0.02 ^A^
	Linoleic acid	y = 22.747x – 7.277	0.992	y = 14.337x^1.157^	0.984	y = 4.076x² + 10.411x − 0.305	1.000	9.39 ± 0.01 ^A^
	Linolenic acid	y = 22.698x – 5.479	0.997	y = 15.859x^1.193^	0.992	y = 2.703x² + 14.366x − 0.576	0.999	9.40 ± 0.04 ^A^
	Cervonic acid	y = 8.810x – 2.972	0.995	y = 5.121x^1.166^	0.958	y = 1.500x² + 4.179x − 0.241	0.999	9.39 ± 0.01 ^A^
TAGs	Tripalmitin	y = 20.345x – 6.634	0.997	y = 12.091x^1.359^	0.995	y = 3.177x² + 10.445x − 0.671	0.999	5.47 ± 0.02 ^C^
	Trilinolein	y = 13.429x – 5.975	0.981	y = 6.424x^1.396^	0.986	y = 3.445x² + 3.030x − 0.134	0.999	5.52 ± 0.01 ^B^

^a^ Data points < 0.5 µg lipid were excluded to fit the linear trend line; ^b^ Average values with corresponding standard deviations of eighteen measurements. Column values with differing letters are significantly different from each other (*p* < 0.05).

**Table 3:** Fatty acid composition (% of total fatty acids) of flours from near-isogenic wheat lines.

	C16:0	C18:0	C18:1	C18:2	C18:3	C20:1
Alpowa Wild-type	20.4 ± 0.1 ^A^	0.9 ± 0.1 ^B^	10.7 ± 0.2 ^B^	64.8 ± 0.3 ^A^	2.8 ± 0.2 ^B^	0.3 ± 0.1 ^A^
Alpowa PINA null	19.5 ± 0.1 ^C^	1.1 ± 0.1 ^B^	11.0 ± 0.2 ^B^	65.3 ± 0.3 ^A^	3.0 ± 0.1 ^B^	0.3 ± 0.1 ^A^
Alpowa PINB G46S	19.5 ± 0.1 ^C^	1.0 ± 0.0 ^B^	11.3 ± 0.5 ^B^	64.8 ± 0.5 ^A^	3.0 ± 0.0 ^B^	0.3 ± 0.1 ^A^
Alpowa PINB W44R	20.3 ± 0.2 ^AB^	1.1 ± 0.1 ^B^	10.8 ± 0.1 ^B^	64.6 ± 0.1 ^A^	2.9 ± 0.1 ^B^	0.3 ± 0.1 ^A^
Svevo	19.5 ± 0.2 ^C^	1.6 ± 0.1 ^A^	14.1 ± 0.2 ^A^	60.9 ± 0.1 ^B^	3.5 ± 0.2 ^A^	0.4 ± 0.1 ^A^
Soft Svevo	19.9 ± 0.1 ^B^	1.6 ± 0.1 ^A^	13.4 ± 0.2 ^A^	61.1 ± 0.1 ^B^	3.5 ± 0.1 ^A^	0.4 ± 0.1 ^A^

Average values with corresponding standard deviations of triplicate measurements are shown. Column values with differing letters are significantly different from each other (*p* < 0.05). C16:0, palmitic acid; C18:0, stearic acid; C18:1, oleic acid; C18:2, linoleic acid; C18:3, α-linolenic acid; C20:1, eicosenoic acid.

## Data Availability

The data presented in this study are available on request from the corresponding author.
